# The genome sequence of *Hypericum pulchrum* L., 1753 (Malpighiales: Hypericaceae)

**DOI:** 10.12688/wellcomeopenres.27278.1

**Published:** 2026-07-30

**Authors:** Raj Whitlock, Robert NL Fitt, Royal Botanic Garden Edinburgh Genome Acquisition Lab, Plant Genome Sizing Collective, Darwin Tree of Life Barcoding collective, Wellcome Sanger Institute Tree of Life Management, Samples and Laboratory team, Wellcome Sanger Institute Scientific Operations: Sequencing Operations, Wellcome Sanger Institute Tree of Life Core Informatics team, Tree of Life Core Informatics collective, Darwin Tree of Life Consortium

**Affiliations:** 1 University of Liverpool, Liverpool, Merseyside, England, UK; 2 Liverpool John Moores University, Liverpool, Merseyside, England, UK

**Keywords:** Hypericum pulchrum, slender St John’s-wort, genome sequence, chromosomal, Malpighiales

## Abstract

We present a genome assembly of *Hypericum pulchrum* (slender St John’s-wort; Streptophyta; Magnoliopsida; Malpighiales; Hypericaceae). The assembly consists of two haplotypes with total lengths of 373.14 megabases and 367.88 megabases. Most of haplotype 1 (94.8%) is scaffolded into 9 chromosomal pseudomolecules. Haplotype 2 was assembled to scaffold level. The mitochondrial sequences have lengths of 217.84 and 276.52 kilobases and the plastid genome assembly has a length of 149.16 kilobases. Gene annotation of this assembly on Ensembl identified 29 497 protein-coding genes. This assembly was generated as part of the Darwin Tree of Life project, which produces reference genomes for eukaryotic species found in Britain and Ireland.

## Species taxonomy

Eukaryota; Viridiplantae; Streptophyta; Streptophytina; Embryophyta; Tracheophyta; Euphyllophyta; Spermatophyta; Magnoliopsida; Mesangiospermae; eudicotyledons; Gunneridae; Pentapetalae; rosids; fabids; Malpighiales; Hypericaceae; Hypericeae; *Hypericum*; *Hypericum pulchrum* L., 1753 (NCBI:txid626748).

## Background

**Figure 1.**
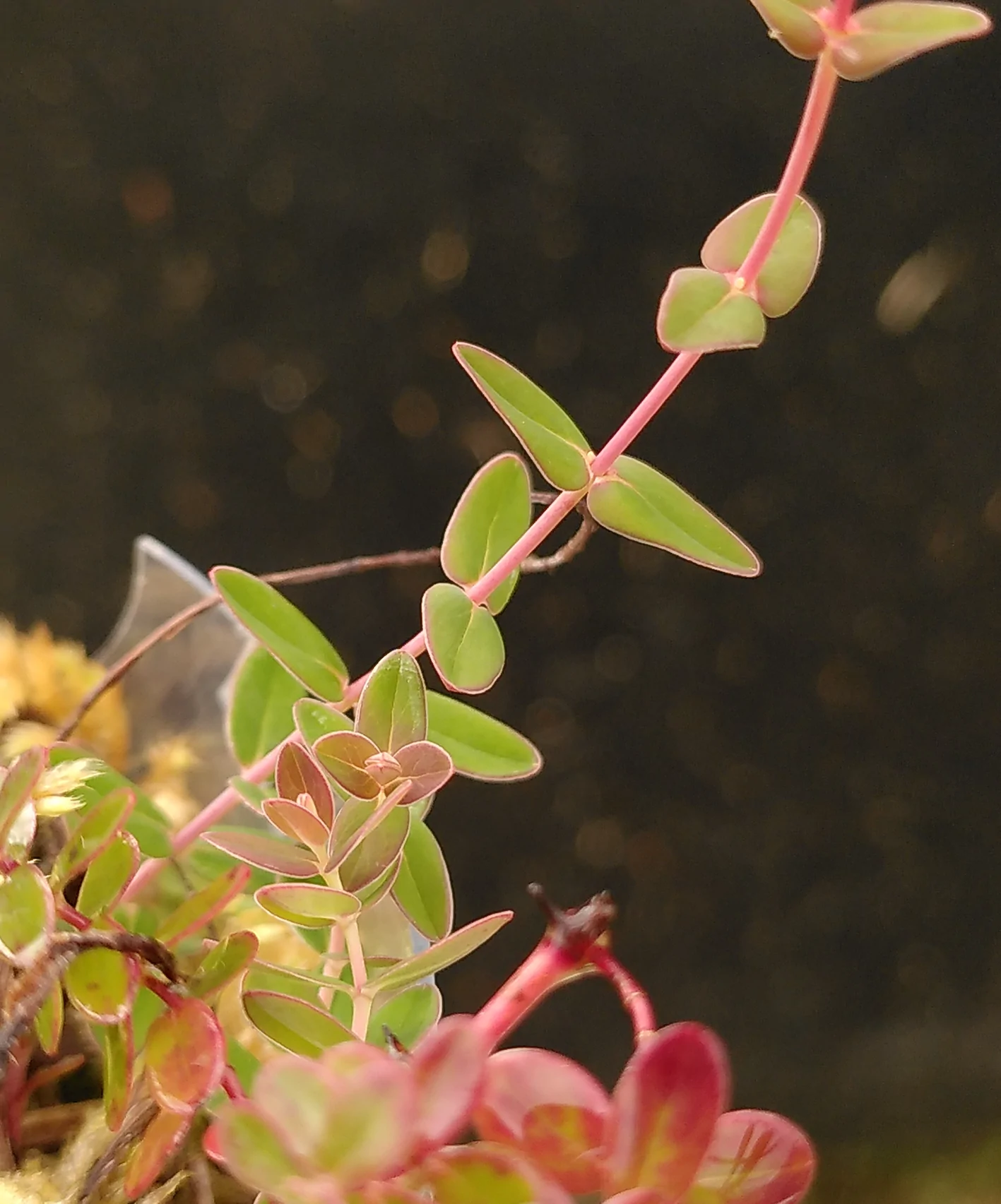
Photograph of the *Hypericum pulchrum* (ddHypPulc1) plant from which samples were taken for genome sequencing.

*Hypericum pulchrum* L. (slender St John’s-wort) is a hairless, erect perennial herb ( Figure 1) found predominantly in dry, non-calcareous and relatively nutrient-poor habitats including heaths, open woodlands and grasslands. This species is widespread across the British Isles and western Europe from Iberia to Norway. The British range occupancy of slender St John’s-wort is the greatest of any native *Hypericum* species, but the species has declined in occurrence in central England with a change index of −0.32 ( Preston *et al.*, 2002; Stroh *et al.*, 2023).

Slender St John’s-wort is typically a minor component of the plant communities in which it occurs. The seed coat of *H. pulchrum* has antifungal properties, which may contribute to the persistence of its seeds in soil, particularly in more alkaline soils ( Basto *et al.*, 2015; Warr *et al.*, 1992). Although slender St John’s-wort is a widespread and often locally abundant native forb, its interactions with phytophagous invertebrates are surprisingly poorly known.

*Hypericum pulchrum* produces a range of biologically-active secondary metabolites, including antioxidant flavonoids and essential oils ( Fico *et al.*, 2006; Nogueira *et al.*, 2008). However, unlike other species in the genus *Hypericum*, slender St John’s-wort is not known to produce hypericin or hyperforin – secondary metabolites with antidepressant properties ( Fico *et al.*, 2006; Ng *et al.*, 2017; Nigutová *et al.*, 2019).

Our specimen of *Hypericum pulchrum* was recovered from a calcareous grassland in Derbyshire, at the Buxton Climate Change Impacts Lab ( Grime *et al.*, 2000). At this site, long-term drought, warming and watering treatments at this site have not driven any significant changes in the abundance of the species ( Fridley *et al.*, 2011). The sampled plant is diploid (2 *n* = 18), consistent with published chromosome counts for the species ( Botanical Society of the British Isles, 2026; Henniges *et al.*, 2022).

Here, we present a high-quality genome sequence for *Hypericum pulchrum.* Since *H. pulchrum* does not produce hyperforin or hypericin, this resource will be valuable for comparative genomics studies to elucidate the biosynthetic pathways for these biomedically important compounds. In addition, the genome sequence could be useful in understanding ecological adaptation to pH, because slender St John’s-wort is one of a subset of British plant species in which both calcicolous and calcifugous populations have evolved.

## Methods

### Sample acquisition, flow cytometry and DNA barcoding

A specimen of *Hypericum pulchrum* (specimen ID EDTOL02194, ToLID ddHypPulc1) was used for genome sequencing. It was collected from Ness Botanic Gardens, Ness, Neston, Cheshire, UK (latitude 53.2726, longitude −3.0463) on 2021-07-14. The specimen was collected and identified by Raj Whitlock and Robert Fitt (University of Liverpool).

The genome size was estimated by flow cytometry following the ‘one-step’ method outlined in Pellicer *et al*. (2021) and using propidium iodide as the fluorochrome. The General Purpose Buffer (GPB) supplemented with 3% PVP was used for isolation of nuclei ( Loureiro *et al.*, 2007), and the internal calibration standard was *Solanum lycopersicum* L. ‘Stupické polní rané’ with an assumed 1C-value of 968 Mb ( Doležel *et al.*, 2007).

The initial identification was verified by an additional DNA barcoding process according to the framework developed by Twyford *et al*. (2024). Part of the plant specimen was preserved in silica gel desiccant ( Chase & Hills, 1991). DNA extracted from the dried plant was amplified by PCR for standard barcode markers, with the amplicons sequenced and compared to public sequence databases including GenBank and the Barcode of Life Database (BOLD) ( Ratnasingham & Hebert, 2007). Following whole genome sequence generation, the relevant DNA barcode region was also used alongside the initial barcoding data for sample tracking at the WSI ( Twyford *et al.*, 2024). The standard operating procedures for Darwin Tree of Life barcoding are available on protocols.io.

### Nucleic acid extraction

Protocols for high molecular weight (HMW) DNA extraction developed at the Wellcome Sanger Institute (WSI) Tree of Life Core Laboratory are available on protocols.io ( Howard *et al.*, 2025). The ddHypPulc1 sample was weighed and triaged to determine the appropriate extraction protocol. For HMW DNA extraction, 101.0 mg of leaf tissue was homogenised by cryogenic bead beating. HMW DNA was extracted using the Plant Organic Extraction protocol. DNA was sheared into an average fragment size of 12–20 kb following the Megaruptor®3 for LI PacBio protocol. Sheared DNA was purified by automated SPRI (solid-phase reversible immobilisation), using AMPure PB beads (Pacific Biosciences) and the Thermo Fisher KingFisher™ Apex to eliminate shorter fragments and concentrate the DNA. The concentration of the sheared and purified DNA was assessed using a Nanodrop spectrophotometer and Qubit Fluorometer using the Qubit dsDNA High Sensitivity Assay kit. Fragment size distribution was evaluated by running the sample on the FemtoPulse system. For this sample, the final post-shearing DNA had a Qubit concentration of 24.4 ng/μL and a fragment size of 16.6 kb. The 260/280 spectrophotometric ratio was 1.75, and the 260/230 ratio was 2.29.

### PacBio HiFi library preparation and sequencing

Library preparation and sequencing were performed at the WSI Scientific Operations core. Libraries were prepared using the SMRTbell Prep Kit 3.0 (Pacific Biosciences) according to the manufacturer’s instructions. The kit includes reagents for end repair/A-tailing, adapter ligation, post-ligation SMRTbell bead clean-up, and nuclease treatment. Size selection and clean-up were performed using diluted AMPure PB beads (Pacific Biosciences). DNA concentration was quantified using a Qubit Fluorometer v4.0 (ThermoFisher Scientific) and the Qubit 1X dsDNA HS assay kit. Final library fragment size was assessed with the Agilent Femto Pulse Automated Pulsed Field CE Instrument (Agilent Technologies) using the gDNA 55 kb BAC analysis kit.

The sample was sequenced on a Revio instrument (Pacific Biosciences, California, USA). Prepared libraries selected for multiplexing were pooled based on genome size, library molarity, and the intended plex level. Primers were annealed and polymerases were bound to generate circularised complexes, following the manufacturer’s instructions. Complexes were purified using SMRTbell beads, diluted to the Revio loading concentration, and spiked with a Revio sequencing internal control. The pooled libraries were sequenced on a Revio 25 M Plex SMRT cell in a 4-plex run. SMRT Link software (Pacific Biosciences), a web-based workflow manager, was used to configure and monitor the run and to carry out primary and secondary data analysis.

### Hi-C

***Sample preparation and crosslinking***

Hi-C data were generated from the leaf tissue of ddHypPulc1 using the Arima-HiC v2 kit (Arima Genomics). Tissue was finely ground using the Covaris cryoPREP Dry Pulverizer (Covaris), and then subjected to nuclei isolation. Nuclei were isolated using a modified protocol based on the Qiagen QProteome Cell Compartment Kit (Qiagen), in which only the Lysis and CE2 buffers were used, with QIAshredder spin columns. After isolation, nuclei were fixed using formaldehyde to a final concentration of 2% to crosslink the DNA. The crosslinked DNA was then digested and biotinylated according to the manufacturer’s instructions. A clean-up step was performed with SPRIselect beads before library preparation. DNA concentration was quantified using the Qubit Fluorometer v4.0 (Thermo Fisher Scientific) and the Qubit HS Assay Kit, following the manufacturer’s instructions.

***Hi-C library preparation and sequencing***

Biotinylated DNA constructs were fragmented using a Covaris E220 sonicator and size selected to 400–600 bp using SPRISelect beads. DNA was enriched with Arima-HiC v2 kit Enrichment beads. End repair, A-tailing, and adapter ligation were carried out with the NEBNext Ultra II DNA Library Prep Kit (New England Biolabs), following a modified protocol where library preparation occurs while DNA remains bound to the Enrichment beads. Library amplification was performed using KAPA HiFi HotStart mix and a custom Unique Dual Index (UDI) barcode set (Integrated DNA Technologies). Depending on sample concentration and biotinylation percentage determined at the crosslinking stage, libraries were amplified with 10–16 PCR cycles. Post-PCR clean-up was performed with SPRISelect beads. Libraries were quantified using the AccuClear Ultra High Sensitivity dsDNA Standards Assay Kit (Biotium) and a FLUOstar Omega plate reader (BMG Labtech).

Prior to sequencing, libraries were normalised to 10 ng/μL. Normalised libraries were quantified again to create equimolar and/or weighted 2.8 nM pools. Pool concentrations were checked using the Agilent 4200 TapeStation (Agilent) with High Sensitivity D500 reagents before sequencing. Sequencing was performed using paired-end 150 bp reads on the Illumina NovaSeq 6000.

### Genome assembly

Prior to assembly of the PacBio HiFi reads, a database of *k*-mer counts ( *k* = 31) was generated from the filtered reads using FastK. GenomeScope2 ( Ranallo-Benavidez *et al.*, 2020) was used to analyse the *k*-mer frequency distributions, providing estimates of genome size, heterozygosity, and repeat content.

The HiFi reads were assembled using Hifiasm in Hi-C phasing mode ( Cheng *et al.*, 2021, 2022), producing two haplotypes. Hi-C reads ( Rao *et al.*, 2014) were mapped to the primary contigs using bwa-mem2 ( Vasimuddin *et al.*, 2019). Contigs were further scaffolded with Hi-C data in YaHS ( Zhou *et al.*, 2023), using the --break option for handling potential misassemblies. The scaffolded assemblies were evaluated using Gfastats ( Formenti *et al.*, 2022), BUSCO ( Manni *et al.*, 2021) and MerquryFK ( Rhie *et al.*, 2020). The organelle genomes were assembled using OATK ( Zhou *et al.*, 2025). The comparator for the mitochondrial assembly was the mitochondrial genome of *Salix dunnii* (NC_058734.1).

### Assembly curation

The assembly was decontaminated using the Assembly Screen for Cobionts and Contaminants ( ASCC) pipeline. TreeVal was used to generate the flat files and maps for use in curation. Manual curation was conducted primarily in PretextView and HiGlass ( Kerpedjiev *et al.*, 2018). Scaffolds were visually inspected and corrected as described by Howe *et al*. (2021). Manual corrections included 23 breaks and 69 joins. This reduced the scaffold count by 11.1% and reduced the scaffold N50 by 46.5%. The curation process is described at sanger-tol/curation-resources . PretextSnapshot was used to generate a Hi-C contact map of the final assembly.

### Assembly quality assessment

The MerquryFK tool ( Rhie *et al.*, 2020) was run in a Singularity container ( Kurtzer *et al.*, 2017) to evaluate *k*-mer completeness and assembly quality for both haplotypes using the *k*-mer database ( *k* = 31) computed prior to genome assembly. The analysis outputs included assembly QV scores and completeness statistics.

The genome was analysed using the BlobToolKit pipeline, a Nextflow implementation of the earlier Snakemake version ( Challis *et al.*, 2020). PacBio reads were aligned to the assembly using minimap2 ( Li, 2018) and processed with SAMtools ( Danecek *et al.*, 2021) to generate coverage tracks. The pipeline runs BUSCO ( Manni *et al.*, 2021) using lineages identified from the NCBI Taxonomy ( Schoch *et al.*, 2020). For the three basal BUSCO lineages, eukaryota, bacteria and archaea, BUSCO genes are aligned to the UniProt Reference Proteomes database ( Bateman *et al.*, 2023) using DIAMOND BLASTp ( Buchfink *et al.*, 2021). The genome assembly is divided into chunks based on the density of BUSCO genes from the closest taxonomic lineage, and each chunk is aligned to the UniProt Reference Proteomes database using DIAMOND BLASTx. Sequences without DIAMOND BLASTx hits are extracted using seqtk and aligned to the NT database using BLASTn ( Altschul *et al.*, 1990). The outputs are consolidated into a BlobDir for visualisation in BlobToolKit. The BlobToolKit pipeline was developed using nf-core tooling ( Ewels *et al.*, 2020) and MultiQC ( Ewels *et al.*, 2016), with containerisation through Docker ( Merkel, 2014) and Singularity ( Kurtzer *et al.*, 2017).

## Genome sequence report

### Sequence data

**Figure 2.**
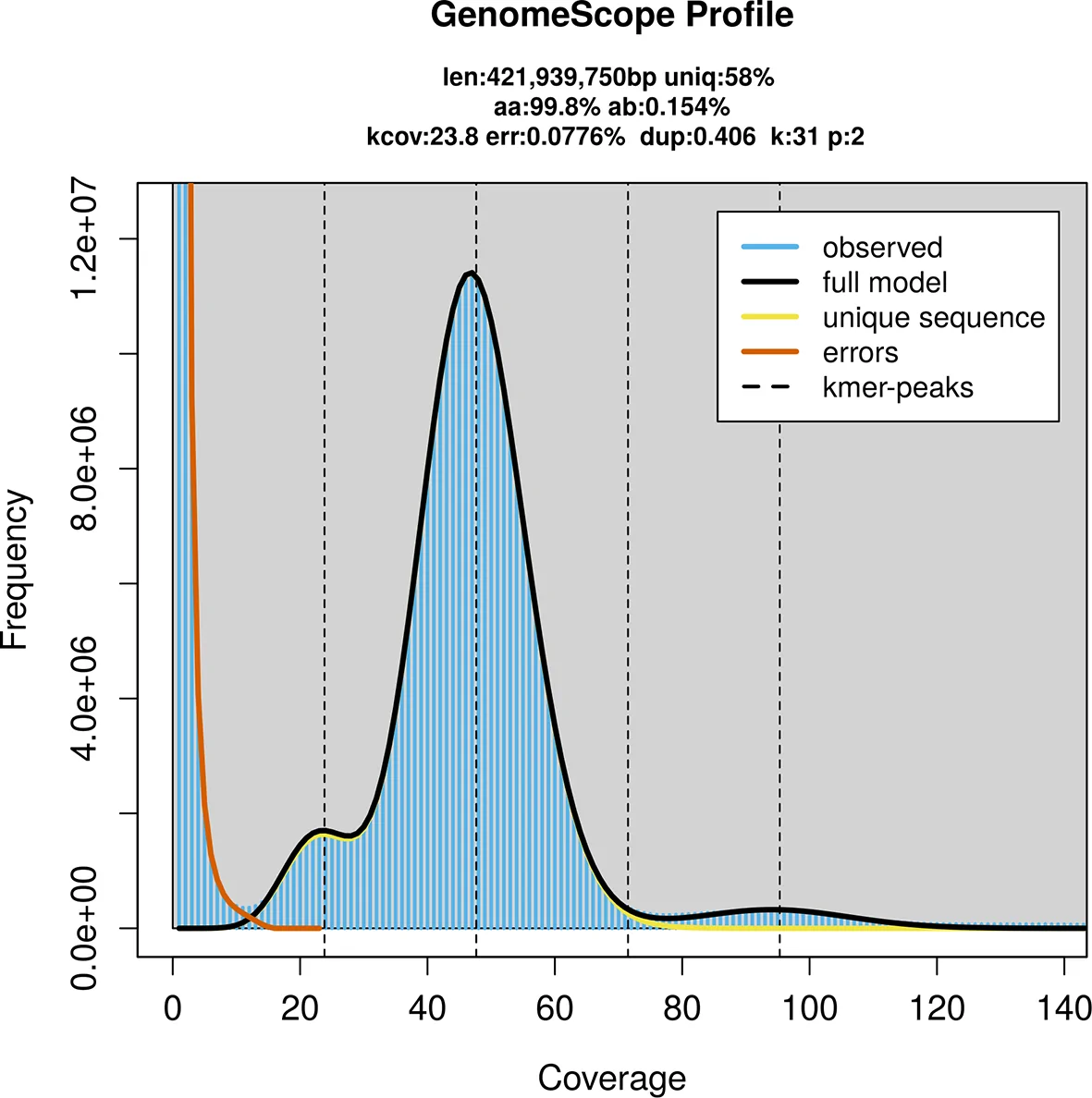
Frequency distribution of *k*-mers generated using GenomeScope2. The plot shows observed and modelled *k*-mer spectra, providing estimates of genome size, heterozygosity, and repeat content based on unassembled sequencing reads.

The genome of a specimen of *Hypericum pulchrum* was sequenced using Pacific Biosciences single-molecule HiFi long reads, generating 20.64 Gb (gigabases) from 1.49 million reads, which were used to assemble the genome. GenomeScope2.0 analysis using a *p* = 2 model gave a *k*-mer-based genome size estimate of 422.24 Mb, with a heterozygosity of 0.15% and repeat content of 42.04% ( Figure 2). Using flow cytometry, the genome size (1C-value) of the sample was estimated to be 0.47 pg, equivalent to 460.00 Mb. These estimates guided expectations for the assembly. Based on the estimated genome size, the sequencing data provided approximately 48× coverage. Hi-C sequencing produced 108.65 Gb from 359.77 million read pairs, which were used to scaffold the assembly. Table 1 summarises the specimen and sequencing details.

**Table 1.** Specimen and sequencing data for *Hypericum pulchrum* (BioProject PRJEB73433).

Platform	PacBio HiFi	Hi-C
**ToLID**	ddHypPulc1	ddHypPulc1
**Specimen ID**	EDTOL02194	EDTOL02194
**BioSample (source individual)**	SAMEA10241452	SAMEA10241452
**BioSample (tissue)**	SAMEA10241645	SAMEA10241644
**Tissue**	leaf	leaf
**Instrument**	Revio	Illumina NovaSeq 6000
**Run accessions**	ERR12721076	ERR12723491
**Read count total**	1.49 million reads	359.77 million read pairs
**Base count total**	20.64 Gb	108.65 Gb

### Assembly statistics

The genome was assembled into two haplotypes using Hi-C phasing. Haplotype 1 was curated to chromosome level, while haplotype 2 was assembled to scaffold level. The final assembly has a total length of 373.14 Mb in 317 scaffolds, with 221 gaps, and a scaffold N50 of 38.88 Mb ( Table 2).

**Table 2.** Genome assembly statistics for *Hypericum pulchrum.*

Genome assembly	Haplotype 1	Haplotype 2
**Assembly name**	ddHypPulc1.hap1.1	ddHypPulc1.hap2.1
**Assembly accession**	GCA_964106755.1	GCA_964106795.1
**Assembly level**	chromosome	scaffold
**Span (Mb)**	373.14	367.88
**Number of chromosomes**	9	-
**Number of contigs**	538	387
**Contig N50**	3.5 Mb	3.46 Mb
**Number of scaffolds**	317	215
**Scaffold N50**	38.88 Mb	38.25 Mb
**Longest scaffold length (Mb)**	43.06	42.38
**Organelles**	Mitochondrial genomes: 217.84 and 276.52 kb; Plastid genome: 149.16 kb	-

The scaffold count follows the NCBI assembly statistics and excludes organelle assemblies, which are reported separately where present.

**Figure 3.**
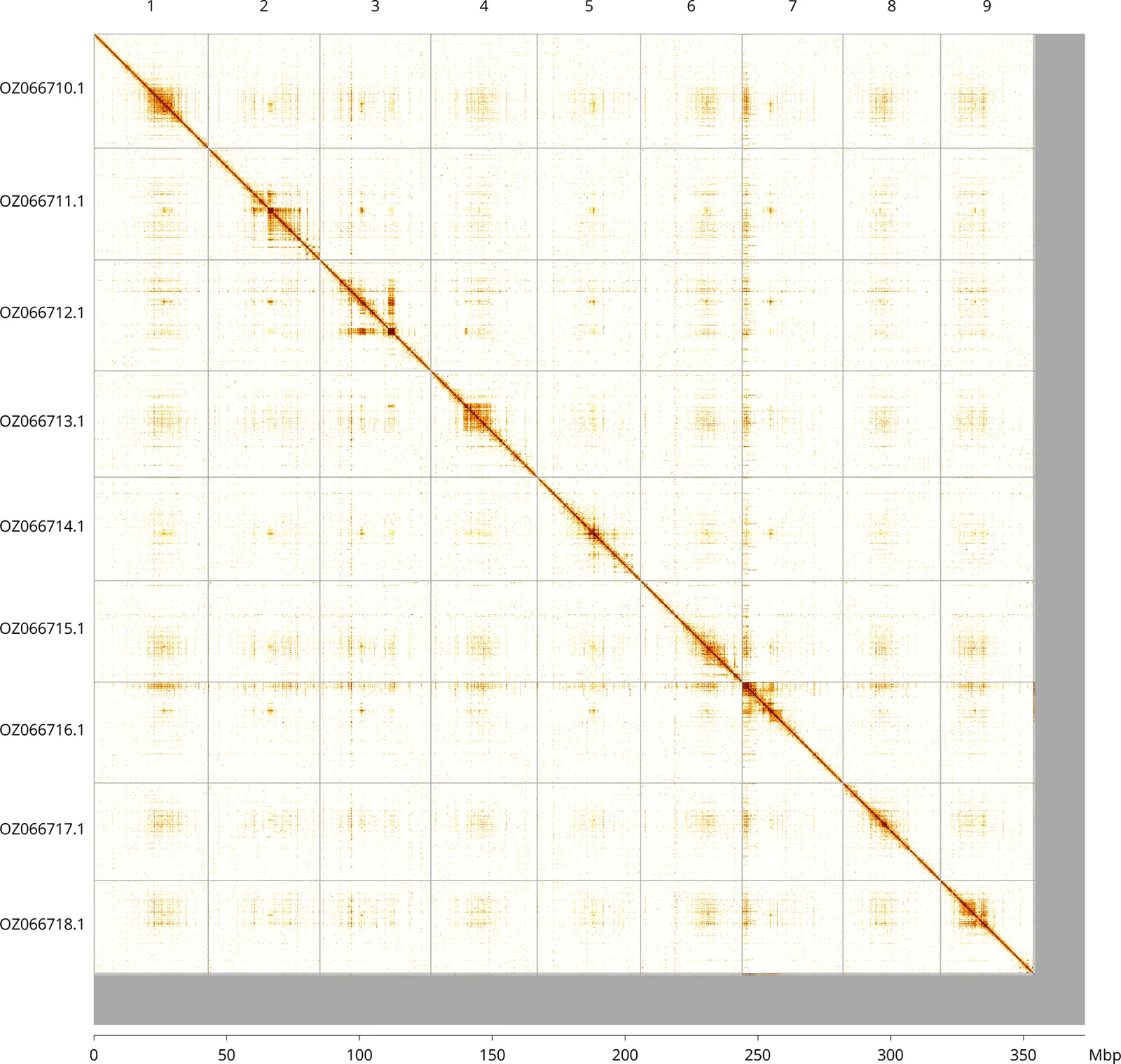
Hi-C contact map of the *Hypericum pulchrum* genome assembly. Assembled chromosomes are shown in order of size and labelled along the axes, with a megabase scale shown below. The plot was generated using PretextSnapshot.

Most of the assembly sequence (94.8%) was assigned to 9 chromosomal-level scaffolds. These chromosome-level scaffolds, confirmed by Hi-C data, are named according to size ( Figure 3; Table 3).

**Table 3.** Chromosomal pseudomolecules in the haplotype 1 genome assembly of *Hypericum pulchrum* ddHypPulc1.

INSDC accession	Molecule	Length (Mb)	GC%
OZ066710.1	1	43.06	38.50
OZ066711.1	2	42.05	38
OZ066712.1	3	41.86	38.50
OZ066713.1	4	40.02	38.50
OZ066714.1	5	38.88	38.50
OZ066715.1	6	38.22	38.50
OZ066716.1	7	38.02	39
OZ066717.1	8	36.70	38.50
OZ066718.1	9	34.92	38.50

Two mitochondrial genomes (lengths 217.84 kb [OZ066719.1] and 276.52 kb [OZ066720.1]) and the plastid genome (length 149.16 kb, OZ066721.1) were also assembled. These sequences are included as contigs in the multifasta file of the genome submission and as standalone records.

### Assembly quality metrics

**Figure 4.**
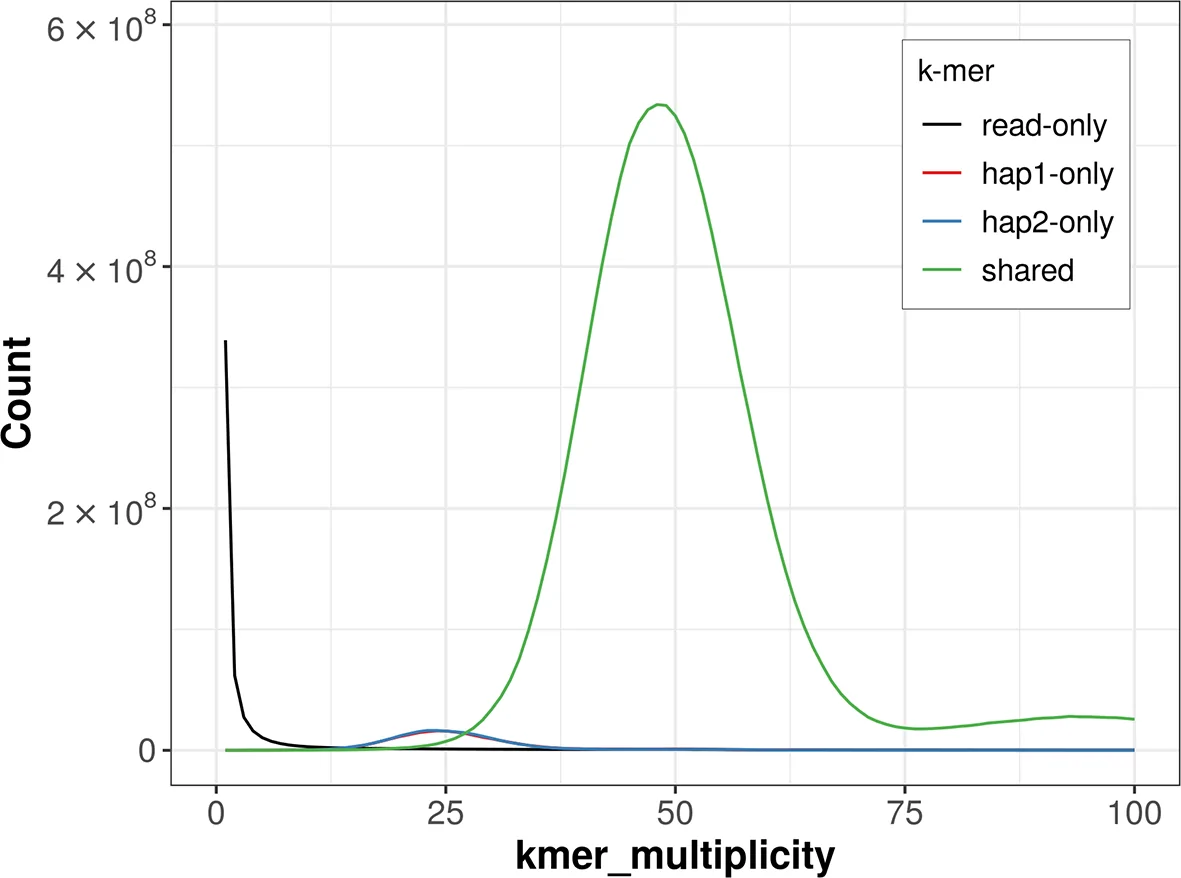
Evaluation of *k*-mer completeness using MerquryFK. This plot illustrates the recovery of *k*-mers from the original read data in the final assemblies. The horizontal axis represents *k*-mer multiplicity, and the vertical axis shows the number of *k*-mers. The black curve represents *k*-mers that appear in the reads but are not assembled. The green curve corresponds to *k*-mers shared by both assemblies, and the red and blue curves show *k*-mers found only in one assembly.

For haplotype 1, the estimated QV is 61.3, and for haplotype 2, 62.0. When the two haplotypes are combined, the assembly achieves an estimated QV of 61.6. The *k*-mer completeness is 95.47% for haplotype 1, 95.54% for haplotype 2, and 99.06% for the combined haplotypes ( Figure 4).

**Figure 5.**
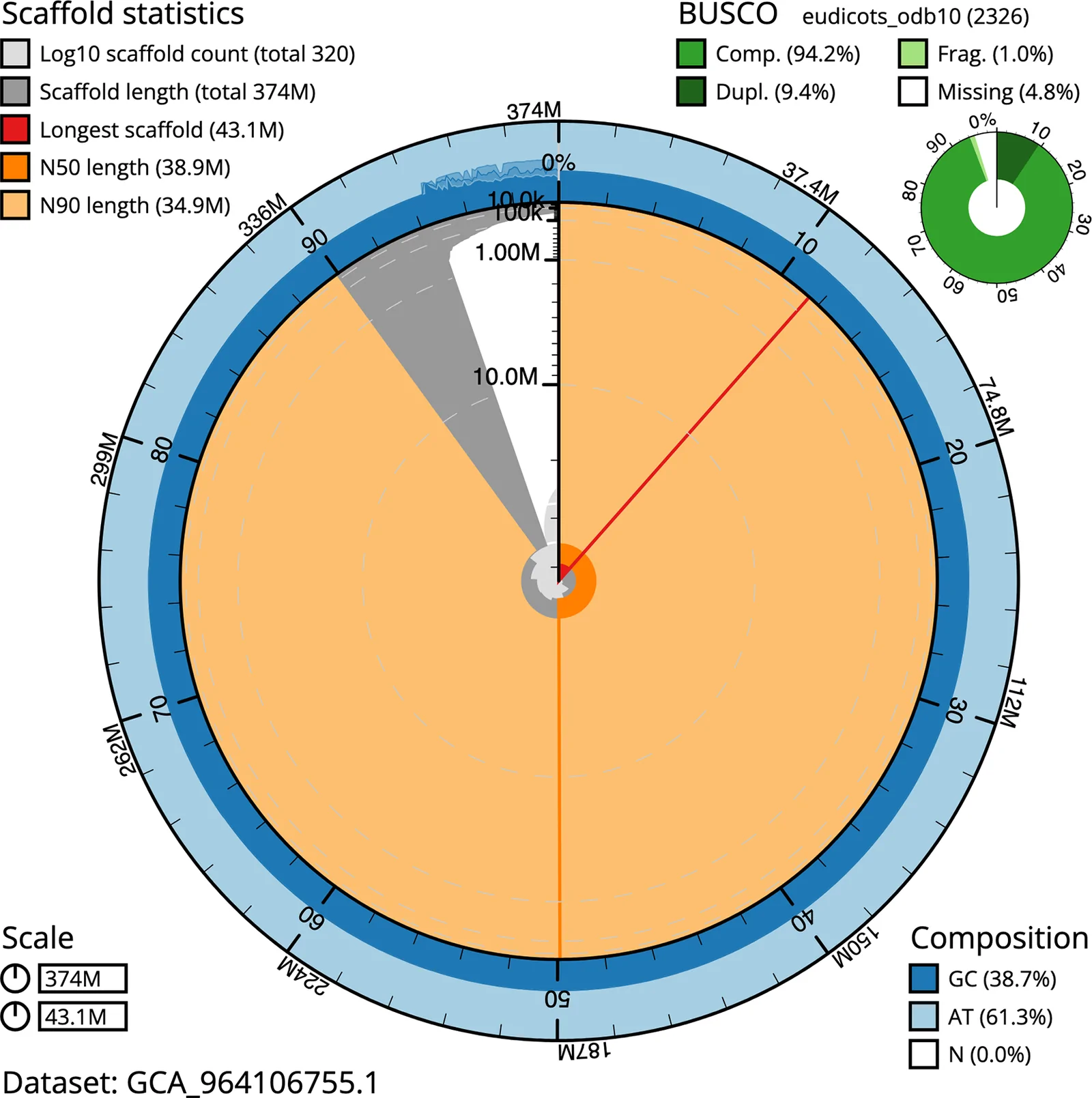
Assembly metrics for ddHypPulc1.hap1.1. The BlobToolKit snail plot provides an overview of assembly metrics and BUSCO gene completeness. The circumference represents the length of the whole genome sequence, and the main plot is divided into 1 000 bins around the circumference. The outermost blue tracks display the distribution of GC, AT, and N percentages across the bins. Scaffolds are arranged clockwise from longest to shortest and are depicted in dark grey. The longest scaffold is indicated by the red arc, and the deeper orange and pale orange arcs represent the N50 and N90 lengths. A light grey spiral at the centre shows the cumulative scaffold count on a logarithmic scale. A summary of complete, fragmented, duplicated, and missing BUSCO genes in the eudicots_odb10 set is presented at the top right. An interactive version of this figure can be accessed on the BlobToolKit viewer.

**Figure 6.**
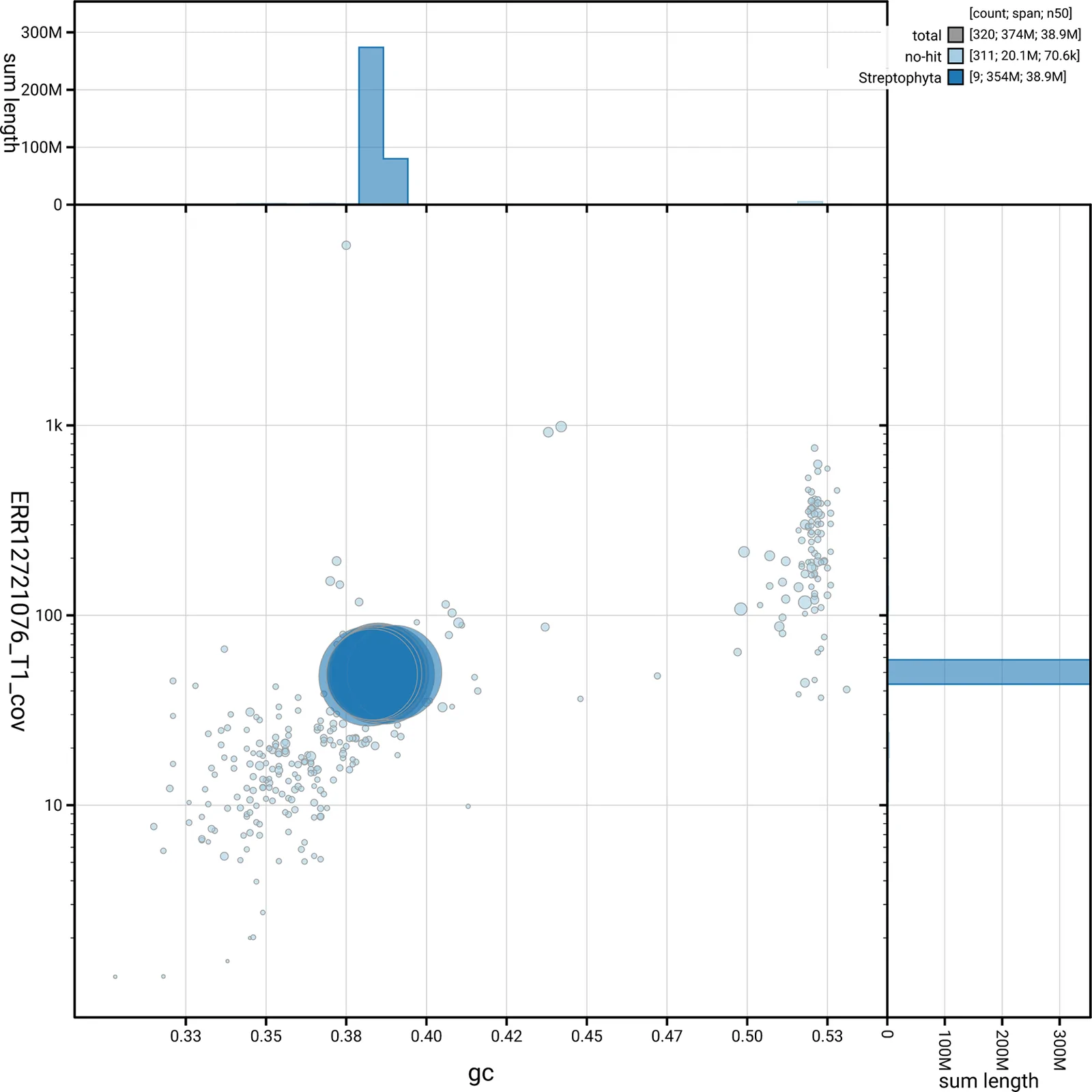
BlobToolKit blob plot for ddHypPulc1.hap1.1. The plot shows base coverage (vertical axis) and GC content (horizontal axis). The circles represent scaffolds, with the size proportional to scaffold length and the colour representing phylum membership. The histograms along the axes display the total length of sequences distributed across different levels of coverage and GC content. An interactive version of this figure is available on the BlobToolKit viewer.

BUSCO analysis using the eudicots_odb10 reference set ( *n* = 2 326) identified 94.2% of the expected gene set (single = 84.8%, duplicated = 9.4%) for haplotype 1. The snail plot in Figure 5 summarises the scaffold length distribution and other assembly statistics for haplotype 1. The blob plot in Figure 6 shows the distribution of scaffolds by GC proportion and coverage for haplotype 1.

Table 4 lists the assembly metric benchmarks adapted from Rhie *et al.* (2021) and the Earth BioGenome Project Report on Assembly Standards January 2026. The EBP metric calculated for haplotype 1 is **6.C.Q61**, meeting the recommended 6.C.Q40 reference standard.

**Table 4.** Earth biogenome project summary metrics for the *Hypericum pulchrum* assembly.

Measure	Value	Benchmark
EBP summary (haplotype 1)	6.C.Q61	6.C.Q40
Contig N50 length	3.50 Mb	≥ 1 Mb
Scaffold N50 length	38.88 Mb	= chromosome N50
Consensus quality (QV)	Haplotype 1: 61.3; haplotype 2: 62.0; combined: 61.6	≥ 40
*k*-mer completeness	Haplotype 1: 95.47%; Haplotype 2: 95.54%; combined: 99.06%	≥ 90%
BUSCO	C:94.2% [S:84.8%, D:9.4%], F:1.0%, M:4.8%, n:2 326	S > 90%; D < 5%
Percentage of assembly assigned to chromosomes	94.80%	≥ 90%

**Notes:** The EBP summary uses log10(Contig N50); chromosome-level (C) or log10(Scaffold N50); Q (Merqury QV). BUSCO: C = complete; S = single-copy; D = duplicated; F = fragmented; M = missing; n = orthologues.

**Table 5.** Software versions and sources used for *Hypericum pulchrum.*

Software	Version	Source
BLAST	2.14.0	ftp://ftp.ncbi.nlm.nih.gov/blast/executables/blast+/
BlobToolKit	4.3.9	https://github.com/blobtoolkit/blobtoolkit
BUSCO	5.5.0	https://gitlab.com/ezlab/busco
bwa-mem2	2.2.1	https://github.com/bwa-mem2/bwa-mem2
DIAMOND	2.1.8	https://github.com/bbuchfink/diamond
fasta_windows	0.2.4	https://github.com/tolkit/fasta_windows
FastK	1.1	https://github.com/thegenemyers/FASTK
GenomeScope2.0	2.0.1	https://github.com/tbenavi1/genomescope2.0
Gfastats	1.3.6	https://github.com/vgl-hub/gfastats
Hifiasm	0.19.8-r603	https://github.com/chhylp123/hifiasm
HiGlass	1.13.4	https://github.com/higlass/higlass
MerquryFK	1.1.0-c1	https://github.com/thegenemyers/MERQURY.FK
Minimap2	2.24-r1122	https://github.com/lh3/minimap2
Oatk	0.9	https://github.com/c-zhou/oatk
MultiQC	1.14; 1.17 and 1.18	https://github.com/MultiQC/MultiQC
Nextflow	23.04.1; 23.10.0	https://github.com/nextflow-io/nextflow
PretextSnapshot	0.0.4	https://github.com/sanger-tol/PretextSnapshot
PretextView	1.0.3	https://github.com/sanger-tol/PretextView
samtools	1.16.1; 1.17; 1.18; 1.19.2; 1.6	https://github.com/samtools/samtools
sanger-tol/ascc	0.1.0	https://github.com/sanger-tol/ascc
sanger-tol/blobtoolkit	0.6.0	https://github.com/sanger-tol/blobtoolkit
sanger-tol/curationpretext	1.4.2	https://github.com/sanger-tol/curationpretext
Seqtk	1.4-r122	https://github.com/lh3/seqtk
Singularity	3.9.0	https://github.com/sylabs/singularity
TreeVal	1.0.2	https://github.com/sanger-tol/treeval
YaHS	1.2a.2	https://github.com/c-zhou/yahs

### Genome annotation report

The *Hypericum pulchrum* genome assembly (GCA_964106755.1) was annotated by Ensembl at the European Bioinformatics Institute (EBI). This annotation includes 66 872 transcribed mRNAs from 29 497 protein-coding and 21 101 non-coding genes. The average transcript length is 2 412.73 bp, with an average of 1.32 coding transcripts per gene and 4.49 exons per transcript. For further information about the annotation, please refer to the annotation page on Ensembl.

## Author information

• Members of the Royal Botanic Garden Edinburgh Genome Acquisition Lab
• Members of the Plant Genome Sizing Collective
• Members of the Darwin Tree of Life Barcoding collective
• Members of the Wellcome Sanger Institute Tree of Life Management, Samples and Laboratory team
• Members of Wellcome Sanger Institute Scientific Operations – Sequencing Operations
• Members of the Wellcome Sanger Institute Tree of Life Core Informatics team
• Members of the Tree of Life Core Informatics collective
• Members of the Darwin Tree of Life Consortium

## Wellcome sanger institute – legal and governance

The materials that have contributed to this genome note have been supplied by a Darwin Tree of Life Partner. The submission of materials by a Darwin Tree of Life Partner is subject to the **‘Darwin Tree of Life Project Sampling Code of Practice’**, which can be found in full on the Darwin Tree of Life website. By agreeing with and signing up to the Sampling Code of Practice, the Darwin Tree of Life Partner agrees they will meet the legal and ethical requirements and standards set out within this document in respect of all samples acquired for, and supplied to, the Darwin Tree of Life Project. Further, the Wellcome Sanger Institute employs a process whereby due diligence is carried out proportionate to the nature of the materials themselves, and the circumstances under which they have been/are to be collected and provided for use. The purpose of this is to address and mitigate any potential legal and/or ethical implications of receipt and use of the materials as part of the research project, and to ensure that in doing so we align with best practice wherever possible. The overarching areas of consideration are:

• Ethical review of provenance and sourcing of the material
• Legality of collection, transfer and use (national and international)

Each transfer of samples is further undertaken according to a Research Collaboration Agreement or Material Transfer Agreement entered into by the Darwin Tree of Life Partner, Genome Research Limited (operating as the Wellcome Sanger Institute), and in some circumstances, other Darwin Tree of Life collaborators.

## Data Availability

European Nucleotide Archive: Hypericum pulchrum (slender St John’s-wort). Accession number PRJEB73433; https://identifiers.org/ena.embl/PRJEB73433. The genome sequence is released openly for reuse. The *Hypericum pulchrum* genome sequencing initiative is part of the Darwin Tree of Life Project (PRJEB40665) andthe Sanger Institute Tree of Life Programme (PRJEB43745). All raw sequence data and the assembly have been deposited in INSDC databases. Raw data and assembly accession identifiers are reported in Tables 1 and 2. Pipelines used for genome assembly at the WSI Tree of Life are available at https://pipelines.tol.sanger.ac.uk/pipelines. Table 5 lists software versions used in this study.
