# Supplementary material for: The genome sequence of Hypericum pulchrum L., 1753 (Malpighiales: Hypericaceae)
Source: Wellcome Open Res. 2026 Jul 30;11:486. [Version 1] doi: 10.12688/wellcomeopenres.27278.1 (PMC13583358; doi:10.12688/wellcomeopenres.27278.1)
Supplement: Peer review history

# Reviewer report: Reviewer response for version 1

**DOI:** 10.21956/wellcomeopenres.30021.r164976

**Published:** 2026-09-16

**Authors:** Oliver Rupp

**Affiliations:** 1 Justus-Liebig-University, Giessengiessen, Germany

This article presents the assembly of *Hypericum pulchrum*. The data creation and data analyses follow state-of-the-art methods for eukaryotic genome assemblies established at the Darwin Tree of Life Consortium, including PacBio Hifi and Hi-C sequencing. The assembly with hifiasm in Hi-C mode was followed by Hi-C scaffolding and manual curation.

 The GenomeScope2 analysis shows three peaks (25x, 50x, 100x). A small 25x peaks could indicate a highly homozygous genome and the 100x peak could result from a genome duplication event. It would be interesting to see the locations of the 25x and 100x regions on the chromosomes (or contigs).

 The methods are clearly presented.

Are sufficient details of methods and materials provided to allow replication by others?

Yes

Is the rationale for creating the dataset(s) clearly described?

Yes

Are the datasets clearly presented in a useable and accessible format?

Yes

Are the protocols appropriate and is the work technically sound?

Yes

Reviewer Expertise:

genome assembly, annotation

I confirm that I have read this submission and believe that I have an appropriate level of expertise to confirm that it is of an acceptable scientific standard.

# Reviewer report: Reviewer response for version 1

**DOI:** 10.21956/wellcomeopenres.30021.r164970

**Published:** 2026-09-12

**Authors:** Peng-Fei Ma

**Affiliations:** 1 Chinese Academy of Sciences, Kunming, China

In this study, the whole genome sequence of *Hypericum pulchrum* was sequenced and assembled. This is a notable plant species occurring in non-calcareous and relatively nutrient-poor habits.   The genome was assembled to the haplotype level, with one of them further scaffolded into chromosomal pseudomolecules. Moreover, evaluation by QV and k-mer completeness indicates a high-quality of the assembled genome, providing valuable genetic resources for studying the evolution of this species.

Are sufficient details of methods and materials provided to allow replication by others?

Yes

Is the rationale for creating the dataset(s) clearly described?

Yes

Are the datasets clearly presented in a useable and accessible format?

Yes

Are the protocols appropriate and is the work technically sound?

Yes

Reviewer Expertise:

Plant genomics and evolution.

I confirm that I have read this submission and believe that I have an appropriate level of expertise to confirm that it is of an acceptable scientific standard.

# Reviewer report: Reviewer response for version 1

**DOI:** 10.21956/wellcomeopenres.30021.r164977

**Published:** 2026-08-21

**Authors:** Panagiotis Madesis

**Affiliations:** 1 University of Thessaly, Volos, Greece

This manuscript presents a high-quality chromosome-scale genome assembly of *Hypericum pulchrum*. Overall, I found the manuscript clear, technically sound, and straightforward to follow. The sequencing, assembly, curation, and quality-assessment approaches are appropriate for a genome resource paper, and the resulting assembly represents a useful addition to the genomic resources available for *Hypericum*.

 The work meets current expectations for a genome data note, particularly in terms of assembly quality and data availability. The conclusions are supported by the results, and I did not identify any major concerns that would prevent indexation. I have, however, identified a small number of points where clarification or additional information would improve the manuscript, particularly with respect to reproducibility and the broader context of the resource.

 The background provides useful ecological, cytological, and phytochemical context for *H. pulchrum* and draws on both established and recent literature. The rationale for generating this genome is also convincing, particularly its potential value for comparative studies of *Hypericum* secondary metabolism, given that *H. pulchrum* is not known to produce hypericin or hyperforin.

 A few minor comments:

- However, the manuscript would benefit from a brief comparison with other *Hypericum* genomes, where available. This would help readers understand how the present assembly adds to existing genomic resources and would place its quality and potential applications in a clearer comparative context.
- A short comparison of assembly contiguity and completeness with other published *Hypericum* genomes would further strengthen the academic context.
- Regarding methodology key parameters for the principal assembly and scaffolding steps could be reported more explicitly, either in the Methods or in supplementary material. A concise record of the relevant command-line options or pipeline configuration would make computational reproduction considerably easier.
- The manual curation step would also benefit from slightly more explanation. The manuscript reports 23 breaks and 69 joins, but the criteria used to determine these corrections are not described in much detail beyond reference to established curation procedures. A brief explanation of how potential misassemblies and joins were identified and accepted would improve transparency.
- Finally, the k-mer analysis gives an estimated genome size of 422.24 Mb, whereas flow cytometry gives an estimate of approximately 460 Mb and haplotype 1 has an assembled span of 373.14 Mb. It would be helpful to include a brief discussion of this difference so that readers can more readily interpret the relationship between the genome-size estimates and the final assembly.

Are sufficient details of methods and materials provided to allow replication by others?

Partly

Is the rationale for creating the dataset(s) clearly described?

Yes

Are the datasets clearly presented in a useable and accessible format?

Yes

Are the protocols appropriate and is the work technically sound?

Yes

Reviewer Expertise:

Plant genetics, genomics, DNA barcoding and metabarcoding, stress tolerance

I confirm that I have read this submission and believe that I have an appropriate level of expertise to confirm that it is of an acceptable scientific standard, however I have significant reservations, as outlined above.
